# IgG4 unveiled: navigating the interplay with Crohn’s disease – from immunology insights to machine learning

**DOI:** 10.1097/MS9.0000000000003633

**Published:** 2025-07-24

**Authors:** Anmol Mohan, Urooj Ghaffar, Ahmad Basharat, Gul Nawaz, Raja Ram Khenhrani, Rimsha, Guillermo de Jesus Aguirre Vera, Priyanka Mohan Lal, Dev Tanush, Muhammad Khuzzaim Khan, Nikhil Duseja, Syeda Laiba Sherazi, Vikash, Vikash Kumar, Dhairya Nanavaty

**Affiliations:** a Karachi Medical and Dental College, Karachi, Pakistan; b Calderdale and Huddersfield NHS Foundation Trust, United Kingdom; c Marshfield Clinic Health System, Wisconsin, United States; d Lyari General Hospital, Karachi, Pakistan; e Ziauddin University, Karachi, Pakistan; f Tecnológico de Monterrey, Mexico; g Dow University of Health Sciences, Karachi, Pakistan; h I.K. Akhunbaev Kyrgyz State Medical Academy, Bishkek, Kyrgyzstan; i Jacobi Medical Center, Albert Einstein College of Medicine, Bronx, New York, United States; j The Brooklyn Hospital Center, New York, United States

**Keywords:** Crohn’s disease, immunoglobulin G4 (IgG4), machine learning

## Abstract

Crohn’s disease (CD) is a chronic inflammatory bowel disease characterized by relapsing-remitting episodes and a progressive course that often leads to bowel damage and disability. While the etiology of CD is multifactorial, involving genetic, environmental, and immunological factors, recent studies have highlighted the role of food antigens and the gut microbiome in its pathogenesis. This paper explores the immunological underpinnings of CD, with a focus on the elevated levels of serum immunoglobulin G4 (IgG4) and their correlation with disease severity and therapeutic response. We review clinical trials and case studies that demonstrate the potential of IgG4-guided exclusion diets and intravenous immunoglobulin (IVIG) therapy in ameliorating CD symptoms and inflammation. Additionally, we delve into advancements in machine learning (ML) models that utilize fecal microbiome data, offering promising diagnostic tools for distinguishing CD from ulcerative colitis and non-IBD conditions. The integration of ML in endoscopy and predictive models for therapy complications signifies a leap toward precision medicine in IBD management. This paper underscores the necessity for a nuanced understanding of CD’s immunological aspects and the innovative application of ML in its diagnosis and treatment, paving the way for personalized therapeutic strategies and improved patient outcomes.

## Introduction

Crohn’s disease (CD) is a chronic inflammatory condition affecting the gastrointestinal tract, marked by symptoms that fluctuate in a relapsing-remitting pattern. It is progressive, leading to bowel damage and resulting in disability. While any part of the gastrointestinal tract can be affected, the terminal ileum and colon are most commonly involved. The inflammation is typically localized, uneven, and penetrates through the entire bowel wall. Although most patients initially present with inflammatory symptoms, about half will eventually develop complications such as strictures, fistulas, or abscesses, often necessitating surgical intervention^[1,2]^. Current therapeutic approaches focus on achieving deep and sustained remission, aiming to prevent complications and halt the disease’s progression^[3]^. Various mechanisms, including direct dietary antigens, alterations in the gut microbiome, and impacts on gastrointestinal permeability, are potential pathways by which food can provoke intestinal inflammation^[4]^. Commercial food hypersensitivity tests, which assess serum IgG4 levels against diverse food antigens, are widely accessible. Recent research has seen a rise in studies on IgG-related adverse reactions to food among individuals with IBD. IgG-related adverse reactions can include digestive issues (such as diarrhea, constipation, bloating, and acid reflux), inflammation (such as rashes, joint pain, and mild swelling), headaches, fatigue, anxiety, and brain fog^[5]^ Common food types that can trigger these reactions include dairy, eggs, peanuts, shellfish, corn, and soy^[6]^. Studies have indicated that patients with IBD exhibit elevated levels of serum IgG4 antibodies to common foods^[7]^. Implementing a food-specific IgG4 antibody-guided exclusion diet has been shown to ameliorate symptoms in individuals with IBD^[8]^.

**HIGHLIGHTS**

- We explored the chronic inflammatory nature of Crohn’s disease, its impact on the gastrointestinal tract, and the progression to complications such as strictures and fistulas.
- We delved into the significance of elevated serum IgG4 levels in Crohn’s disease patients, examining how these levels correlate with disease severity and response to treatment.
- The paper highlighted the therapeutic potential of IgG4-guided exclusion diets in alleviating symptoms and reducing inflammation in Crohn’s disease.
- We showcased the advancements in machine learning algorithms using fecal microbiome data for the accurate diagnosis of Crohn’s disease and differentiation from other inflammatory bowel diseases.
- The paper also discussed the innovative application of artificial intelligence in endoscopy to enhance the assessment and management of inflammatory bowel disease.
- We presented machine learning models that predict therapy complications and the likelihood of initiating biologic therapy in Crohn’s disease patients.

In another study, it was found that levels of serum IgG4 directed against certain food antigens were notably elevated in patients with IBD compared to healthy controls^[9]^. This trend was more prevalent among patients with CD than those with ulcerative colitis (UC), potentially correlating with the severity of the disease. Elevated levels of IgG4 targeting food antigens may suggest heightened exposure of antigens to the mucosal immune system, possibly due to increased mucosal permeability, which could contribute to the onset or worsening of IBD. An IgG4-guided exclusion diet has been shown to alleviate symptoms and reduce inflammation in patients with CD^[10]^. Similarly, nutritional interventions based on food-specific IgG serum levels have been reported to significantly reduce stool frequency and abdominal pain in CD patients compared to a placebo diet^[11]^. Immunological adverse reactions mediated by IgG antibodies seem to exacerbate damage and increase permeability, but these effects may be reversed through exclusion diets^[12]^. In CD, the persistence of fibrosis may be attributed to the involvement of localized IgG4-related fibroplasia. IgG4-related disease (IgG4RD) is characterized by widespread fibrosis, which can manifest in various forms, including autoimmune pancreatitis, cholangitis, aortitis, retroperitoneal fibrosis, and salivary gland fibrosis^[13]^. Current understanding of IgG4RD pathophysiology suggests that T-cell-mediated inflammation triggers excessive production of IgG4 antibodies and systemic fibroplasia^[14,15]^. While IgG4 antibodies are believed to possess anti-inflammatory properties, indicating they may not be directly pathogenic but rather reactive to an upstream process, the exact nature of the inflammation initiating this disease remains challenging to characterize^[16]^. One proposed mechanism is an autoimmune response to self-antigens, although its validity across the spectrum of IgG4RD remains uncertain^[17–19]^. Transparent and standardized reporting is critical when discussing novel methodologies and their implications in clinical research, particularly those involving artificial intelligence and immunological biomarkers [TITAN 2025]^[20]^ (Table 1).

**Table 1:** TITAN guideline checklist

Section	Item	Description	Reported in manuscript	Page/section
**Title & Abstract**	1	Clearly identify AI use in the title or abstract	Yes	Title, Abstract
**Introduction**	2	Justify the use of AI within the clinical or research context	Yes	Introduction (final paragraph)
**Methods – AI System**	3	Describe the AI system/model (name, version, developer, source)	No	Not specified
	4	Specify whether the AI is a diagnostic, predictive, or analytic tool	Yes	ML in Diagnosis section
	5	Report data input types and sources used by the AI system	Yes	ML and Fecal Microbiome section
	6	Describe training, validation, and test datasets used	No	Not applicable (review article)
**Methods – Clinical Use**	7	Describe how AI was integrated into clinical decision-making	No	Not applicable (no clinical AI use)
	8	Describe human-AI interaction or oversight	No	Not applicable
**Outcomes & Evaluation**	9	Define primary and secondary outcomes evaluated	Yes	Abstract, Methods
	10	Specify performance metrics for AI (AUC, accuracy, etc.)	Yes (partially)	ML section – model accuracy stated
**Ethics & Reproducibility**	11	Mention ethical approval and consent (if applicable)	Yes	Noted as N/A – review article
	12	Describe measures for reproducibility (e.g., code/data sharing)	No	Not applicable
**Limitations**	13	Discuss AI-related limitations and interpretability	Yes	Conclusion, ML limitations noted
**Declaration of AI Use**	14	Declare use of AI tools (e.g., ChatGPT, for writing/analysis)	No	Should be added if used
**Checklist Compliance**	15	Submit completed TITAN checklist	Yes	This document

## Methods

Our review offers a comprehensive examination of Crohn’s disease, particularly focusing on the impact of an IgG4-exclusion diet and intravenous immunoglobulin in alleviating CD symptoms and inflammation. We also delve into the emerging role of machine learning, which leverages fecal microbiome data to provide promising diagnostic tools for distinguishing Crohn’s disease from ulcerative colitis and non-IBD conditions.

To ensure a thorough exploration of the subject, we developed a search strategy using key terms such as “Crohn’s disease,” “Machine Learning,” “Fecal Microbiome Data,” “Immunoglobulin G4 (IgG4),” and “Intravenous Immunoglobulin.” This search string was applied across multiple databases, including PubMed, Google Scholar, ClinicalTrials.gov, Scopus, and Embase, to identify relevant studies. After a rigorous review of the identified articles, we extracted and analyzed key data for inclusion in our review.

## Immunological basis of Crohn’s disease

**Figure 1.**
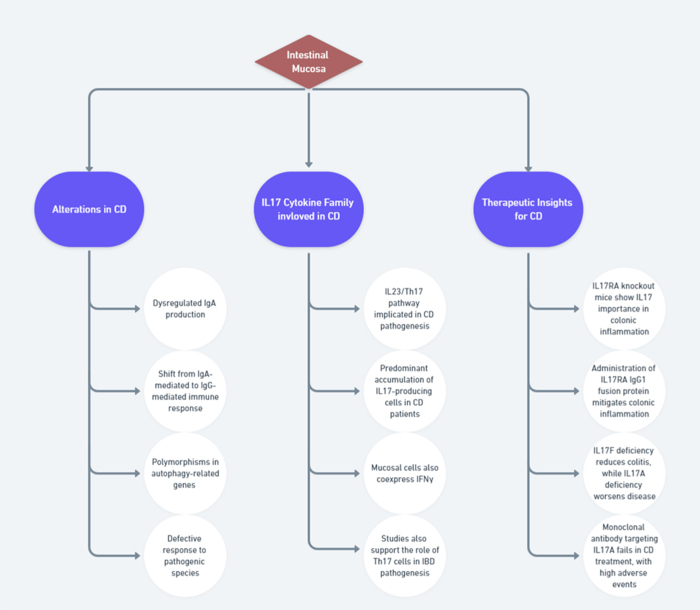
Immunological basis for Crohn’s disease.

The intestinal mucosa comprises various components, including epithelial cells, goblet cells, Paneth cells, stromal cells, and immune cells. The epithelial layer, composed of a single layer of tightly connected cells, contains immune cells interspersed among them and forms structures such as villi and crypts of Lieberkühn^[21]^. This layer plays a dual role, facilitating nutrient absorption and serving as a physical barrier against luminal antigens. Goblet cells secrete mucus, while Paneth cells release antimicrobial peptides, both limiting microbial spread^[22]^ (Fig. 1). In Crohn’s disease (CD), a decrease in goblet cell numbers is associated with a thinner mucus layer^[23]^, while abnormal mucus composition is more typical of ulcerative colitis (UC)^[24]^.

Beneath the epithelium, the lamina propria contains stromal cells such as fibroblasts, myofibroblasts, and pericytes that mediate wound healing and fibrosis, partly by secreting chemokines like CCL19, CCL21, and interleukin (IL)-33^[25]^. Altered immune homeostasis is a hallmark of CD. Immunoglobulin A (IgA) is produced by B cells in the intestinal lamina propria and is crucial for mucosal immunity. Dendritic cells regulate IgA production in Peyer’s patches through microbial recognition^[26]^. IgA helps maintain microbial balance, and disruptions can shift mucosal immunity toward an immunoglobulin G (IgG)-mediated response^[27,28]^.

Notably, recent findings suggest that some CD patients exhibit increased production of immunoglobulin G4 (IgG4), the least abundant IgG subclass, typically associated with anti-inflammatory or tolerogenic functions. Elevated IgG4 may result from chronic antigen exposure and Th2-biased responses. Unlike other IgG subclasses, IgG4 does not fix complement and may instead function to dampen inflammation. Although its exact role in CD remains unclear, some studies have reported elevated serum IgG4 levels in CD patients and infiltration of IgG4-positive plasma cells in intestinal tissue. This raises the possibility that IgG4 could either represent a regulatory countermeasure to ongoing inflammation or contribute to immune dysregulation. Its presence may serve as a marker of chronic immune stimulation or a unique disease phenotype.

Another key immune defense mechanism, autophagy, also appears to be disrupted in CD. Polymorphisms in autophagy-related genes, such as *ATG16L1* (autophagy-related 16-like 1), *IRGM* (immunity-related GTPase M), *ULK1, PTPN2*, and *LRRK2*, have been associated with increased disease susceptibility^[29]^. Since *NOD2* recruits *ATG16L1* during pathogen sensing, risk variants in either gene may impair the autophagic response to intracellular bacteria^[30,31]^. CD patients homozygous for risk variants in *ATG16L1* exhibit structural abnormalities in Paneth cells, including reduced granules and lysosomes^[32]^. These defects compromise antimicrobial peptide secretion, potentially leading to uncontrolled bacterial growth and chronic inflammation^[33]^.

The IL-17 cytokine family, particularly IL-17A, the major product of Th17 cells, plays a critical role in CD pathogenesis. Alongside IL-17A, Th17 cells also produce IL-21, IL-22, interferon-γ (IFNγ), and tumor necrosis factor (TNF)^[34]^. Activation of the IL-23/Th17 pathway is central to CD immunopathology^[35]^. Studies have identified a buildup of IL-17-producing cells in the submucosa and muscularis propria of CD patients^[36,37]^. Notably, some IL-17-producing T cells also coexpress IFNγ and can be polarized toward a Th1 phenotype under IL-12 stimulation, suggesting functional plasticity^[38,39]^.

Animal studies provide additional insight. In a trinitrobenzene sulfonic acid (TNBS)-induced colitis model, *Il17ra*-deficient mice were protected from colitis, and administration of an IL-17RA fusion protein ameliorated inflammation^[40]^. In contrast, dextran sulfate sodium (DSS)-induced models showed that *Il17f* deficiency reduced inflammation, while *Il17a* deficiency worsened it^[41]^. These mixed results were reflected in clinical trials, where secukinumab (anti-IL-17A) failed to demonstrate benefit in CD and was associated with worsened disease^[42]^ (Table 2).

**Table 2:** Studies exploring the role of immunoglobulins in Crohn’s disease

Study	Patients/case description	Treatment	Outcome
Raedler *et al*^[38]^	20 patients with pre-treatment Crohn’s disease activity index (CDAI) ranging from 200 to 300.	- Steroids and aminosalicylates- IVIG treatment with IgM-enriched IVIG (Pentaglobin, Biotest AG, Germany)	IVIG group: Greater mean decrease in CDAINon-IVIG group: Lesser decrease in CDAICRP count decreased significantly in IVIG groupNo adverse effects reported
Rohr *et al*^[39]^	Case of a patient with symptoms consistent with CD and a CDAI of 312.	Unspecified dosage of IVIG (Venimmun, Behring AG, Germany) for 7 days	Significant pain relief by Day 2CDAI decreased to 70 after 5 weeksNormal stools confirmed at 2 and 5 weeks post-treatmentNo signs of active inflammation at 5 weeks
Wolf *et al*^[40]^	Case study of a patient with over 4 years of symptoms (bloody diarrhea, fever, and weight loss) and steroid resistance.	7-day course of IVIG (Sandoglobulin, Sandoz AG, Switzerland) at a dosage of 0.4 g/kg/d	Improvement after 2 daysComplete resolution of symptoms after 5 daysRelapses successfully treated with IVIGAdditional relapses required steroids
Raedler *et al*^[41]^	Ten patients with a mean CDAI of 150 received 4 days of treatment with IgM-enriched IVIG (Pentaglobin, Biotest AG, Germany) in addition to steroid and sulfasalazine medication.	IVIG (Pentaglobin, Biotest AG, Germany) + steroid and sulfasalazine medication	Significant improvement in CDAI and other parametersNo reported side effects
Sorensen and Kallick^[42]^	Case of a steroid-resistant 12-year-old patient with colonoscopy-verified inflamed ileum.	IVIG therapy at a dosage of 1 g/kg/d for 3 days	Clinical improvement within 3 daysMonthly IVIG infusions enabled discontinuation of steroid dosage without side effects
Possoz *et al*^[43]^	Six steroid-dependent patients with CD underwent IVIG treatment.	IVIG treatment	Remission without relapses over 33 ± 3 weeksSome patients experienced transient headaches, shivers, and fever after fifth round of treatment
Schmidt^[44]^	Three steroid-resistant patients with CD responded well to a 10-day course of IVIG (Venimmun, Behring AG, Germany).	10-day course of IVIG (Venimmun, Behring AG, Germany)	Decreased pain, diarrhea, and improved serological indicationsNo side effects observed
Knoflach *et al*^[45]^	Six CD patients treated with IVIG.	IVIG treatment	Half showed reduced CDAI at 16 weeksTwo remained in steroid-free remission at 18 and 21 monthsSome patients did not maintain response to IVIG treatment
Rohr *et al*^[46]^	24 CD patients received a 7-day course of IVIG (Venimmun, Behring AG, Germany).	7-day course of IVIG (Venimmun, Behring AG, Germany)	Significant improvements in CDAI15 patients considered treatment successesAlleviation of extra-intestinal symptomsNo improvement observed in fistulae
Teuber *et al*^[47]^	Seven CD patients treated with a 7-day course of IVIG (Venimmun, Behring AG, Germany).	7-day course of IVIG (Venimmun, Behring AG, Germany)	Sharp decrease in CDAI in six patients over 4–8 weeksRemission experienced by three newly-diagnosed patients at 90 days post-treatment

## Machine learning in the diagnosis of Crohn’s disease and IgG4-related disease

Recent advances in machine learning (ML) have enhanced diagnostic capabilities in immune-mediated diseases, including CD and IgG4-related disease (IgG4-RD)^[43]^. While many ML studies analyze CD and IgG4-RD separately, the two diseases can share overlapping gastrointestinal symptoms, fibrosis, and systemic inflammation, posing diagnostic challenges^[44]^.

In CD, ML algorithms have been trained on radiographic data (e.g., CT enterography, MRI), histopathology, and genomics to improve diagnostic accuracy, disease classification, and treatment response prediction^[45]^. Similarly, ML has facilitated the identification of IgG4-RD using features from imaging, serology (elevated IgG4), and histopathology. Given the potential overlap in clinical presentation and tissue involvement (e.g., bowel thickening, lymphoplasmacytic infiltrate), ML could be instrumental in distinguishing CD from IgG4-RD, particularly in ambiguous cases^[46]^. Future ML applications may include multi-modal integration of radiology, tissue IgG4 staining, and molecular profiles to stratify disease subtypes and improve early diagnosis.

## Evidence for elevated IgG4 levels in Crohn’s disease

Given the possible role of IgG4 in CD, attention has turned toward treatments that modulate immunoglobulin pathways. Intravenous immunoglobulin (IVIG), particularly preparations enriched with IgG and IgM, has shown therapeutic potential in immune-mediated diseases.

Raedler *et al*^[43]^ conducted a non-randomized trial involving 20 patients with a pre-treatment Crohn’s Disease Activity Index (CDAI) ranging from 200 to 300. Half of the participants received steroids and amino salicylates, while the other half received additional intravenous immunoglobulin (IVIG) treatment with IgM-enriched IVIG (Pentaglobin, Biotest AG, Germany). The IVIG group showed a greater mean decrease in CDAI compared to the non-IVIG group, which experienced a lesser decrease over the same period. The C-reactive protein (CRP) levels dropped from 3.5 mg/dL to 1.5 mg/dL over 14 days in the IVIG group, compared to a decrease from 3.7 mg/dL to 1.9 mg/dL in the control group. No adverse effects were reported, though the study did not specify the clinical benefits associated with these reductions in various markers.

Rohr *et al*^[44]^ reported a case involving a patient with symptoms consistent with Crohn’s Disease (CD) and a CDAI of 312. The patient received an unspecified dosage of IVIG (Venimmun, Behring AG, Germany) for 7 days. By Day 2, the patient experienced pain relief, and the CDAI decreased to 70 after 5 weeks of treatment. The patient reported no further abdominal pain and had normal stools at 2- and 5-week post-treatment. A follow-up colonoscopy at 5 weeks showed no signs of active inflammation, and no relapses or side effects were observed within 5 weeks post-treatment. Two other patients with a CDAI over 300 also showed significant improvement after receiving identical treatment, but further details were limited.

Wolf *et al*^[45]^ presented a case study of a patient with over 4 years of symptoms, including bloody diarrhea, fever, and weight loss, who was resistant to steroids. Despite previous unsuccessful treatments with prednisolone, cortisone enemas, metronidazole, and sulfasalazine, a 7-day course of IVIG (Sandoglobulin, Sandoz AG, Switzerland) at a dosage of 0.4 g/kg/d led to improvement after 2 days, with complete resolution of symptoms after 5 days. The patient experienced two relapses after the initial treatment, each successfully treated with IVIG within 3 to 6 days. However, additional relapses required steroids for remission.

Raedler *et al*^[46]^ discussed a study involving ten patients with a mean CDAI of 150 who received 4 days of treatment with IgM-enriched IVIG (Pentaglobin, Biotest AG, Germany) in addition to steroid and sulfasalazine medication. Evaluation on Day 14 showed significant improvement in CDAI and other parameters compared to the control group, with no reported side effects (Table 3).

**Table 3:** Important key points from the review

Section	Key information
**Introduction**	CD is a persistent inflammatory condition affecting the GI tract, often involving the terminal ileum and colon. Involves dysregulated immune responses, including IgA to IgG shifts and autophagy-related gene mutations. IgG4 levels and the impact of IgG4-guided diets. IgG4-related disease (IgG4RD) associated with fibrosis and various autoimmune conditions.
**Immunological basis of Crohn’s disease**	Intestinal mucosa includes epithelial cells, goblet cells, Paneth cells, and stromal cells. Abnormalities in IgA production, autophagy-related gene mutations, and IL17 cytokine involvement. IL17-producing cells and Th17 pathway play a role in CD pathogenesis.
**Evidence for elevated IgG4 levels in Crohn’s disease**	IVIg treatment has shown potential in reducing CDAI and symptoms. Studies show mixed results, with some patients achieving significant improvement and others experiencing transient side effects. Elevated IgG4 levels noted in some patients with CD, suggesting potential therapeutic targets.
**Machine Learning in Diagnosis**	ML models using blood, urine, and fecal biomarkers show high accuracy in diagnosing IBD. AI and ML techniques help in predicting therapy complications and personalizing treatment. Fecal microbiome data has shown promise in differentiating between IBD types and improving diagnostic accuracy.
**Therapeutic Implications**	IVIg acts through immunomodulation and may help rebalance immune responses. The mechanism involves Fc-receptor blockade, modulation of cytokine responses, and disruption of complement aggregation. Further research is needed to refine IVIg protocols and understand its full therapeutic potential.
**Future Directions**	Need for standardized clinical trials and protocols for IVIg therapy. Focus on elucidating IVIg’s immune-modulating pathways and exploring personalized therapies. Integration of machine learning, collaborative research, and translational studies to optimize IVIg’s clinical utility.

Sorensen and Kallick^[47]^ described the case of a steroid-resistant 12-year-old patient with a colonoscopy-verified inflamed ileum. IVIG therapy at a dosage of 1 g/kg/d for 3 days led to clinical improvement within 3 days, and monthly IVIG infusions allowed for the discontinuation of steroid dosage without side effects.

Possoz *et al*^[48]^ reported on six steroid-dependent patients with CD who underwent IVIG treatment, resulting in remission without relapses over 33 ± 3 weeks. However, four patients experienced transient headaches, shivers, and fever after the fifth round of treatment, leading to treatment cessation.

Schmidt^[49]^ described three steroid-resistant patients with CD who responded well to a 10-day course of IVIG (Venimmun, Behring AG, Germany), experiencing decreased pain, diarrhea, and improved serological indications. No side effects were observed.

Knoflach *et al*^[50]^ reported on six CD patients treated with IVIG, with half showing reduced CDAI at 16 weeks, and two remaining in steroid-free remission at 18 and 21 months. However, some patients did not maintain their response to IVIG treatment, with CDAI and serum alpha protein levels rising after 2 weeks.

Rohr *et al*^[51]^ summarized a study in which 24 CD patients received a 7-day course of IVIG (Venimmun, Behring AG, Germany). Significant improvements in CDAI were reported, with 15 patients considered treatment successes. Extra-intestinal symptoms were alleviated in some patients, though no improvement was observed in fistulae. No side effects were reported.

Teuber *et al*^[52]^ discussed seven CD patients treated with a 7-day course of IVIG (Venimmun, Behring AG, Germany), resulting in a sharp decrease in CDAI in six patients over 4–8 weeks. Three patients with extra-intestinal symptoms experienced remission, although there was no improvement in existing fistulae. Three newly-diagnosed patients remained in remission at 90 days post-treatment, with no reported side effects.

## Machine learning in the diagnosis of Crohn’s disease and IgG4-related disease

A recent study demonstrated that a machine learning prediction model, utilizing readily available blood, urine, and fecal biomarkers alongside patient age, can effectively support the diagnosis of both ulcerative colitis and Crohn’s disease with high accuracy^[53]^. Given the multifaceted nature of inflammatory bowel disease (IBD), encompassing its pathogenesis and therapeutic strategies, artificial intelligence (AI) has emerged as a promising tool. AI can enhance the understanding of IBD etiology, assist in diagnosing and stratifying IBD patients, and facilitate the integration of precision medicine into clinical settings^[54]^.

Moreover, the application of AI in endoscopy, particularly AI-driven endoscopy in IBD, shows significant potential for improving the assessment of IBD. However, further research is necessary to fully realize its benefits^[55,56]^. Additionally, machine learning techniques have proven useful in identifying the risk of therapy complications in IBD. For instance, McDonell *et al* demonstrated how Random Forest (RF) regression models could identify elevated CRP levels and longer disease duration as predictors of hyperglycemia in IBD patients undergoing intravenous glucocorticosteroid treatment^[57]^. Similarly, Choi *et al* developed a machine learning model to forecast the five-year probability of initiating biologic therapy in IBD patients^[58]^.

Recent advancements in machine learning (ML) models utilizing fecal microbiome data have shown significant promise in diagnosing IBD. For example, a study employing Operational Taxonomic Units (OTUs) in conjunction with the Random Forest algorithm achieved high accuracy in distinguishing IBD from non-IBD groups. Additionally, it was successful in differentiating between ulcerative colitis (UC) and Crohn’s disease (CD)^[59]^. In another study, various feature selection techniques were applied to build an RF model that made significant progress in diagnosing UC and CD separately^[60]^. Furthermore, an RF model developed using taxonomic profiles at the species level achieved excellent diagnostic results for UC and CD^[61]^. Another study employed the supervised Partial Least Squares Discriminant Analysis (sPLS-DA) model, which showed exceptional performance in diagnosing IBD and distinguishing between UC and CD, statistically surpassing previous studies^[62]^. These advancements in utilizing fecal microbiome data to develop ML models hold great potential for improving the diagnosis of IBD.

The application of machine learning in diagnosing IgG4-related disease is also gaining traction. Machine learning models can analyze complex datasets, including imaging, histopathological, and serological data, to identify patterns and biomarkers specific to IgG4-related disease. For instance, a study demonstrated the use of a machine learning algorithm to differentiate IgG4-related disease from other similar conditions with high accuracy, utilizing features such as serum IgG4 levels and imaging findings. These advancements highlight the potential of machine learning to enhance the diagnostic accuracy and understanding of IgG4-related disease, paving the way for more personalized treatment approaches^[63]^.

The validity of using AI in diagnosing IBD is supported by multiple studies demonstrating high accuracy, sensitivity, and specificity in differentiating IBD from non-IBD conditions. However, challenges remain, including technical barriers, bias within data sets, and the need for validation in larger, diverse cohorts. Despite these obstacles, the potential for AI to transform clinical management of IBD is significant, with ongoing research aimed at refining these models for broader clinical application^[64]^.

## Therapeutic implications

The mechanism of action of immunoglobulins (Ig) in Crohn’s disease (CD) is likely complex, given their successful application across a wide range of diseases. Primarily, immunoglobulins function through replacement and/or immunomodulation, although this distinction is somewhat limited in the context of CD. Variable production of Ig, including cases of common variable immunodeficiency, has been associated with CD, where immunoglobulin replacement may be crucial for affected patients. The role of Ig as an immunomodulator is less clear, with several proposed mechanisms including Fc-receptor blockade, induction of B-cell anergy, or a broader anti-inflammatory effect. While the mechanism is undoubtedly multifactorial, reduced T-cell proliferation and trafficking appear to be the most physiologically plausible mechanisms in CD. This reduced trafficking, mediated by intravenous immunoglobulin (IVIg), has also been documented in ulcerative colitis (UC)^[65]^.

IVIg, derived from pooled immunoglobulin G (IgG) from numerous healthy blood donors, is administered intravenously and has been utilized for both approved and off-label treatments of various immune deficiencies, autoimmune diseases, acute infections, and chronic inflammatory conditions^[66,67]^. It has been suggested that certain chronic inflammatory disorders may result from inadequate immune responses, and IVIg administration could rebalance the immune response, favoring the host over the pathogen^[9]^. The primary proposed mechanism involves altering the expression and function of macrophage Fc receptors^[68]^. Understanding the balance between inhibitory and activating Fc receptors is crucial for comprehending immune function and regulation^[69,70]^. Additionally, IVIg infusion may suppress pathogenic cytokines, modulate B and T cell responses, neutralize harmful autoantibodies, and disrupt complement aggregation at target tissues^[71,72]^.

## Future directions

The existing literature on intravenous immunoglobulin (IVIg) in Crohn’s disease (CD) largely consists of anecdotal reports, with a notable lack of controlled trials. The absence of standardized criteria for usage, dosing, concurrent therapies, and other variables restricts the generalizability of the findings across different studies.

Future research in Crohn’s disease should focus on several key areas. Clinical trials are essential to establish standardized protocols for IVIg therapy, including dosing regimens and concurrent therapies, to enhance the comparability and generalizability of results. Long-term studies are needed to evaluate IVIg’s efficacy and potential adverse effects, as well as to investigate its immune-modulating mechanisms in CD. Additionally, efforts should be directed toward biomarker discovery and patient stratification to enable personalized IVIg therapy. Exploring combination therapies and integrating machine learning for predictive modeling represent promising avenues for advancing treatment strategies.

Translational research that bridges basic science with clinical practice, patient-centered outcomes research, health economic analyses, and collaborative data-sharing initiatives are crucial for optimizing IVIg’s clinical utility in CD and improving patient care. These approaches will help in refining treatment protocols and enhancing overall patient outcomes.

## Conclusion

Crohn’s disease (CD) presents a complex interplay of immunological dysregulation and gastrointestinal inflammation. Recent research highlights the potential therapeutic role of intravenous immunoglobulin (IVIg) in alleviating symptoms and reducing inflammation in CD patients. While existing studies offer promising outcomes, the lack of standardized protocols and controlled trials limits the generalizability of findings. Moving forward, future directions in CD research should prioritize conducting clinical trials to establish standardized IVIg protocols, elucidating IVIg’s immune-modulating pathways, and exploring personalized therapy approaches based on biomarker discovery and patient stratification. Integration of machine learning for predictive modeling, collaborative data-sharing initiatives, and translational research bridging basic science and clinical practice will be crucial for optimizing IVIg’s clinical utility and enhancing patient care in CD management.

## Data Availability

Not applicable.
